# Phase I randomized dose-ascending placebo-controlled trials of ferroquine - a candidate anti-malarial drug - in adults with asymptomatic *Plasmodium falciparum *infection

**DOI:** 10.1186/1475-2875-10-53

**Published:** 2011-03-01

**Authors:** Ghyslain Mombo-Ngoma, Christian Supan, Matthias P Dal-Bianco, Michel A Missinou, Pierre-Blaise Matsiegui, Carmen L Ospina Salazar, Saadou Issifou, Daniel Ter-Minassian, Michael Ramharter, Maryvonne Kombila, Peter G Kremsner, Bertrand Lell

**Affiliations:** 1 Medical Research Unit, Albert Schweitzer Hospital, Lambaréné, Gabon; 2 Institute of Tropical Medicine, University of Tübingen, Germany; 3 Département de Parasitologie-Mycologie, Faculté de Médecine, Université des Sciences de la Santé, Libreville, Gabon; 4 Sanofi-Aventis Research and Development, Chilly-Mazarin, France; 5 Department of Medicine I, Division of Infectious Diseases and Tropical Medicine, Medical University of Vienna, Austria

## Abstract

**Background:**

The development and spread of drug resistant *Plasmodium falciparum *strains is a major concern and novel anti-malarial drugs are, therefore, needed. Ferroquine is a ferrocenic derivative of chloroquine with proven anti-malarial activity against chloroquine-resistant and -sensitive *P. falciparum *laboratory strains.

**Methods:**

Adult young male aged 18 to 45 years, asymptomatic carriers of *P. falciparum*, were included in two-dose escalation, double-blind, randomized, placebo-controlled Phase I trials, a single dose study and a multiple dose study aiming to evaluate oral doses of ferroquine from 400 to 1,600 mg.

**Results:**

Overall, 54/66 patients (40 and 26 treated in the single and multiple dose studies, respectively) experienced at least one adverse event, 15 were under placebo. Adverse events were mainly gastrointestinal symptoms such as abdominal pain (16), diarrhoea (5), nausea (13), and vomiting (9), but also headache (11), and dizziness (5). A few patients had slightly elevated liver parameters (10/66) including two patients under placebo. Moderate changes in QTc and morphological changes in T waves were observed in the course of the study. However, no adverse cardiac effects with clinical relevance were observed.

**Conclusions:**

These phase I trials showed that clinically, ferroquine was generally well-tolerated up to 1,600 mg as single dose and up to 800 mg as repeated dose in asymptomatic young male with *P. falciparum *infection. Further clinical development of ferroquine, either alone or in combination with another anti-malarial, is highly warranted and currently underway.

## Background

*Plasmodium falciparum *has become resistant to several commonly used anti-malarial drugs. As a consequence new anti-malarial compounds are urgently needed. Despite a considerable increase in international efforts in anti-malarial drug development, there is at present a shortage of truly novel drugs which do not share the same mechanisms of action and drug resistance with those that are failing today. The entire reliance on artemisinin derivatives in the drug development over the past decade might be deceptive in the light of first reports of artemisinin resistant *P. falciparum *[1].

Ferroquine (SSR97193) - a ferrocenic derivative of chloroquine and thus a 4-aminoquinoline compound - is a truly novel anti-malarial candidate drug. Ferroquine demonstrated anti-malarial activity in vitro and in vivo [2-5]. In vitro, ferroquine has a potent activity on *Plasmodium falciparum *laboratory strains, whether they are sensitive or resistant to chloroquine. In vivo, four days of treatment with ferroquine efficiently cured mice infected with either chloroquine-resistant or sensitive strains of *Plasmodium vinckei*. Ferroquine is a racemic compound with both enantiomers showing an anti-malarial activity similar to that of the parent compound, in vitro and in vivo. Ferroquine is metabolized into one major metabolite (N-monodemethylated), also highly active in vitro. Tests on field isolates confirmed the activity of ferroquine against parasites that are multi-resistant to most marketed anti-malarial agents and did not show any significant cross sensitivity with major anti-malarials currently used [5-8].

### Preclinical studies

Ferroquine was tested in preclinical studies prior to its use in humans, alone or in combination with artesunate, showing it to be devoid of relevant adverse effects on central nervous system, respiratory, renal, and gastrointestinal functions. The only notable effects of ferroquine alone, or in combination with artesunate, were observed on cardiovascular function. In vitro ferroquine, at a high concentration, was shown to affect the action potential pattern as a result of human ether-à-go-go related gene (hERG) channel inhibition (inhibitory concentration decreasing a response by 50% (IC50) = 907 ng/mL). In vivo, in anaesthetized but not in conscious dogs, ferroquine induced from blood concentrations ≥200 ng/mL major haemodynamic effects. In vivo, in conscious dogs, a ferroquine/artesunate combination (at 30 mg/kg and 100 mg/kg per os, respectively, i.e., ferroquine blood concentrations of 669 ng/mL) was devoid of effects on blood pressure, heart rate, PR interval, and QRS complex duration whereas QTcB and QTcF were slightly increased [artesunate alone had no significant effect on cardiovascular and electrocardiogram parameters]. However, no increases in QT, QTc were noted in the 13-week oral toxicity study in dogs at 0.2/25, 1/25 and 5/25 mg/kg of ferroquine/artesunate combination and at 5 mg/kg of ferroquine alone.

### Previous human experience

First, in human studies of ferroquine were conducted in a double-blind, randomized, placebo controlled, and sequential dose escalation study in 42 healthy male subjects in Germany [unpublished data]. Single oral doses up to 800 mg were clinically well tolerated and no drug-related clinically relevant adverse event was observed. One serious adverse event occurred which was unrelated to the study drug and all other adverse events were mild to moderate and no relevant electrocardiogram abnormalities, such as QT prolongation or repolarisation abnormalities were described.

This is a report on two clinical phase I trials, which aimed to assess the clinical, and laboratory safety and tolerability profile of ferroquine. These studies were designed to evaluate ascending oral doses in young male volunteers with asymptomatic *P. falciparum *infection and to determine the maximum tolerated dose after oral administration of ferroquine.

## Methods

### Study design, settings and population

Both clinical trials were designed as phase I, randomized, double blind, placebo-controlled, sequential dose escalation studies. The first study TDU5967 ("Single Dose Study") was designed as a dose escalation trial of a single oral dose, whereas the second study TDR5969 ("3 Day Repeated Dose Study") aimed to evaluate a three-day treatment course.

Clinical work was carried out from April 2005 to June 2006 at the Medical Research Unit of the Albert Schweitzer Hospital in Lambaréné, Gabon, a semi-urban town of about 30,000 inhabitants where malaria transmission is perennial with moderate seasonal variation [9,10].

Male volunteers aged between 18 and 45 years residing in Lambaréné and its surrounding areas were invited to participate. Individuals having provided written informed consent and harbouring asymptomatic *P. falciparum *infection were enrolled into the study if they fulfilled the following criteria: (1) weight between 50 and 90 kg, (2) BMI of 18 to 28 kg/m^2^, (3) normal vital signs after 10 minutes resting in supine position, i.e. systolic blood pressure between 95 and 140 mmHg, and diastolic blood pressure between 50 and 90 mmHg, and heart rate between 45 and 90 beats per minute, (4) normal 12-lead automatic ECG, (5) and no fever or history of fever in the last 72 hours. Exclusion criteria were (1) presence of any clinical signs of malaria, (2) previous anti-malarial treatment, (3) significant co-morbidities or other conditions impeding compliance with the study, (4) presence or history of drug allergy, (5) evidence for hepatitis B, C, or HIV infection (HBs antigen, anti-HCV antibodies, anti-HIV1&2 antibodies).

The studies were granted ethical clearance by the Ethics Committee of the International Foundation of the Albert Schweitzer Hospital. The study protocols complied with recommendations of the 18^th ^World Health Congress (Helsinki, 1964) and all applicable amendments, also with laws, regulations, and any applicable guideline of Gabon, the country where the studies were conducted in compliance with the International Harmonized Conference on Good Clinical Practices guidelines. Informed consent was obtained prior to the conduct of any study-related procedures.

### Interventions

#### Randomization and blinding

Study treatments were prepared by dose level for each patient according to the study randomization list. The following doses of ferroquine were to be explored, 400 mg, 800 mg, 1,200 mg, 1,400 mg, and 1,600 mg for the single dose study and 400 mg, 600 mg, 800 mg, and 1,000 mg for the three-day repeated dose study.

Treatment allocation was performed in sequential groups of eight participants per dose group. Six participants were to receive ferroquine and two participants were to receive placebo. All ferroquine dose strengths and placebo capsules were identically matched. The first patients were assigned to the lowest dose level of the study.

The treatment allocation was double-blinded and was done according to the randomization list which was computer-generated. The allocation code was concealed at site and procedures were put in place to have the option for code breaking in case of urgent clinical need. Decision for dose escalation was based on a thorough review of clinical and laboratory data, including ECG and pharmacokinetic data of the preceding dose level.

### Study procedures

#### Screening and drug administration

Recruitment of volunteers was started with a pre-screening visit to determine the *P. falciparum *infection status by blood smear, Rapid Test and PCR [11]. If infection was confirmed by at least one out of these three laboratory tests, the volunteers were invited to undergo screening procedure, including a complete physical examination, haematology and biochemistry analysis, urinalysis, urine drug screen, and viral serology (HIV, HBSAg, HCV). Eligible volunteers were admitted as inpatients to the Medical Research Unit and study drugs were administered orally with noncarbonated water under fasted conditions. Treatment duration was one day in the single dose study and three days in the multiple dose study. Volunteers were discharged from the hospital two days after the last drug intake and were followed-up for eight weeks in both studies.

#### Recording and reporting of adverse events and cardiac effects

Adverse events were defined as any clinical change from baseline or previous visit spontaneously reported by the patient or observed by the investigator. All adverse events regardless of seriousness or relationship to the investigational product were recorded. Adverse events were coded according to Medical Dictionary for regulatory Activities (MedDRA, version 8.1).

Due to the very long half-life of the product, around 20 days, the rule applied for the emergence of adverse event was 5 half-lives after administration. Thus, an adverse event was considered as emergent until the end-of-study visit. Abnormalities of vital signs, laboratory parameters or ECG readings were recorded as adverse events only if they were medically relevant by being symptomatic, requiring corrective treatment, leading to discontinuation and/or fulfilling a seriousness criterion.

A standard 12-lead ECG was performed hourly up to six hours post-dosing, two-hourly up to 12 hours post-dose, and weekly until the end of follow-up. ECG measures were obtained from measurement of three consecutive QRS complexes and averaged. Heart rates, QTc (Bazett and Fridericia) were derived from the mean values of the measured parameters. In addition, a 24-hr ECG was recorded in the repeated dose study for five days to detect cardiac rhythm abnormalities.

### Outcomes and statistics

Primary outcomes (endpoints) for these randomized clinical trials were defined as the clinical and laboratory adverse event profile and the effect of ferroquine on the cardiovascular system.

Sample size for these studies was based upon empirical considerations from clinical phase I studies and no formal sample size calculation was performed. Safety analysis was based on participants who were administered at least one dose of study drug (exposed population) including participants who withdrew consent prematurely. If a participant had received the study drug and prematurely stopped study participation he was replaced, unless if follow up data were available up to day 15.

## Results

### Study population

**Figure 1:**
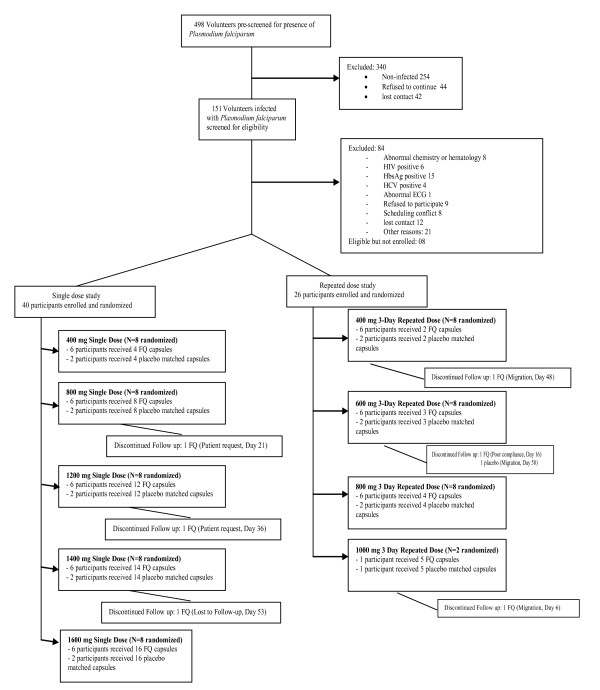
**Flow of participants**.

Out of a total of 498 volunteers pre-screened, 158 persons with *P. falciparum *infections were further screened for eligibility. Eighty-four were excluded because of abnormal chemistry or haematology results, abnormal ECGs, or other health problems. Seventeen volunteers could not be enrolled due to logistical reasons and further eight volunteers were not enrolled because the predefined number of participants for each of the two studies was already reached. Recruitment and participant flow are depicted in Figure 1.

A total of 66 asymptomatic male volunteers infected with *P. falciparum *were enrolled, randomized, and treated. In the single dose study, a total of 30 volunteers were exposed to single oral doses of ferroquine over a range from 400 mg to 1,600 mg and 10 received placebo. In the three-day multiple dose study, 19 volunteers were treated for three days with daily doses from 400 mg to 1,000 mg and seven received placebo. Only one participant was administered the 1,000 mg dose. Enrolment was discontinued at 1,000 mg dose because of morphologic changes in T waves and a moderate QT prolongation observed in patients treated with 800 mg and 1,000 mg. Seven participants stopped the study prematurely for reasons unrelated to adverse events, three in the single dose study and four in the three-day repeated dose study (Figure 1). Baseline characteristics of study participants are provided in Table 1 and Table 2.

**Table 1:** Demographic and clinical characteristics of participants at baseline in the single dose study

Parameters (Unit)	Statistics/Category	Single Dose Study					
		
		Placebo	Ferroquine				
			
		(N = 10)	400 mg (N = 6)	800 mg (N = 6)	1200 mg (N = 6)	1400 mg (N = 6)	1600 mg (N = 6)
**Age (years)**	Mean (SD)	30.5 (9.4)	32.0 (4.4)	31.2 (6.1)	30.5 (8.8)	24.3 (6.3)	26.5 (4.0)
	
	Min - Max	18 - 44	27 - 37	22 - 38	20 - 42	18 - 34	19 - 30

**Height (cm)**	Mean (SD)	167.3 (8.7)	168.8 (7.0)	168.7 (3.9)	166.2 (7.4)	172.7 (6.7)	164.3 (5.0)
	
	Min - Max	147 - 176	160-176	163-174	154-174	163 - 183	157 - 172

**Weight (kg)**	Mean (SD)	66.0 (7.8)	62.8 (9.3)	64.7 (3.6)	62.7 (8.5)	65.2 (3.5)	60.6 (5.2)
	
	Min - Max	55 - 78	50-71	58-59	52-75	61 - 70	57 - 71

**BMI (kg/m^2^)**	Mean (SD)	23.59 (2.40)	21.92 (1.85)	22.77 (1.61)	22.65 (1.83)	21.92 (1.25)	22.48 (0.88)
	
	Min - Max	20.1-27.6	19.5-24.3	19.8-24.5	19.6-24.8	19.7 - 23.0	21.9 - 24.0

**BSA (m^2^)**	Mean (SD)	1.74 (0.14)	1.72 (0.16)	1.74 (0.05)	1.70 (0.15)	1.74 (0.14)	1.78 (0.08)
	
	Min - Max	1.5 - 1.9	1.5 - 1.8	1.7 - 1.8	1.5 - 1.9	1.5 - 1.9	1.7 - 1.9

**HR (bpm)**	Mean (SD)	50.9 (6.9)	56.3 (3.7)	54.4 (5.9)	53.3 (9.3)	56.8 (6.6)	54.6 (9.3)
	
	Min - Max	42 - 67	51 - 61	46 - 63	41 - 62	47 - 66	44 - 66

**QTc B (ms)**	Mean (SD)	374.7 (19.6)	378.9 (20.6)	374.0 (11)	368.3 (16.9)	369.4 (28.9)	371.3 (20.6)
	
	Min - Max	347 - 405	346 - 405	362 - 392	337 - 388	327 - 408	334 - 396

**QTc F (ms)**	Mean (SD)	386.5 (22.1)	384.0 (19.3)	380.5 (9.3)	376.9 (13.5)	373.4 (26.7)	378.4 (20.8)
	
	Min - Max	357 - 416	350 - 403	363 - 389	356 - 391	341 - 416	353 - 409

NOTE: BMI: Body Mass Index; BSA: Body Surface Area; HR: Heart Rate; QTc (Bazett Fridericia); N: Count of patients; SD: standard deviation; Min: minimum; Max: maximum

**Table 2:** Demographic and clinical characteristics of participants at baseline in the multiple dose study

Parameters (Unit)	Statistics/Category	Multiple Dose Study				
		
		Placebo	Ferroquine			
			
		(N = 7)	400 mg (N = 6)	600 mg (N = 6)	800 mg (N = 6)	1000 mg (N = 1)
**Age (years)**	Mean (SD)	23.6 (5.3)	30.2 (6.2)	26.2 (6.3)	24.5 (5.8)	NA
	
	Min - Max	18 - 33	25 - 40	18 - 35	19 - 32	

**Height (cm)**	Mean (SD)	168.6 (6.5)	167.5 (5.8)	166.5 (10.5)	170.8 (9.9)	NA
	
	Min - Max	160 - 178	158 - 173	150 - 181	155 - 184	

**Weight (kg)**	Mean (SD)	63.0 (2.6)	64.8 (8.3)	59.8 (10.1)	61.3 (5.0)	NA
	
	Min - Max	59 - 66	55 - 75	50 - 74	56 - 70	

**BMI (kg/m^2^)**	Mean (SD)	22.24 (1.61)	23.03 (1.74)	21.48 (1.97)	21.05 (1.19)	NA
	
	Min - Max	20.5 - 25.0	21.3 - 25.3	19.2 - 24.5	19.8 - 23.3	

**BSA (m^2^)**	Mean (SD)	1.67 (0.09)	1.73 (0.13)	1.66 (0.19)	1.72 (0.13)	NA
	
	Min - Max	1.6 - 1.8	1.5 - 1.9	1.4 - 1.9	1.5 - 1.9	

**HR (bpm)**	Mean (SD)	53.8 (6.9)	59.2 (7.8)	55.6 (3.7)	52.9 (1.2)	NA
	
	Min - Max	48 - 63	52 - 72	51 - 60	48 - 62	

**QTc B (ms)**	Mean (SD)	366.6 (11)	380.7 (15.7)	359.0 (21.8)	373.8 (24)	NA
	
	Min - Max	358 - 384	367 - 409	150 - 181	340 - 406	

**QTc F (ms)**	Mean (SD)	373,9 (6.3)	382.0 (11.7)	362.8 (22.5)	381.2 (24.5)	NA
	
	Min - Max	368 - 382	372 - 403	335 - 404	340 - 399	

NOTE: BMI: Body Mass Index; BSA: Body Surface Area; HR: Heart Rate; QTc (Bazett Fridericia); N: Count of patients; SD: standard deviation; Min: minimum; Max: maximum

### Frequency of adverse events

Details on the frequency of adverse events by dose-group are depicted in Table 3 and Table 4. In the single, ascending dose study, most patients in the placebo group (9/10) presented at least one adverse event during the study versus 3/6 patients in the 400 m dose group, 1/6 in the 800 mg dose group, 6/6 in the 1,200 mg and 1,400 mg dose groups, and 4/6 patients in the 1,600 mg dose group.

**Table 3:** Frequency of the most common adverse events during the single dose study

System organ class	Preferred term	Single Dose Study					
		
		Placebo	Ferroquine				
			
		(N = 10)	400 mg (N = 6)	800 mg (N = 6)	1200 mg (N = 6)	1400 mg (N = 6)	1600 mg (N = 6)
**Any class**	Any TEAE	9 (90)	3 (50)	1 (17)	6 (100)	6 (100)	4 (67)

**Gastrointestinal disorders**	Any TEAE	4 (40)	0	0	3 (50)	4 (67)	4 (67)
	
	Abdominal pain	3 (30)	0	0	0	3 (50)	2 (33)
	
	Diarrhoea	0	0	0	1 (17)	2 (33)	0
	
	Nausea	1 (10)	0	0	1 (17)	1 (17)	2 (33)
	
	Toothache	1 (10)	0	0	1 (17)	0	0
	
	Vomiting	0	0	0	1 (17)	1 (17)	2 (33)

**Nervous system disorders**	Any TEAE	2 (20)	0	0	3 (50)	1 (17)	3 (50)
	
	Dizziness	1 (10)	0	0	0	1 (17)	2 (33)
	
	Headache	1 (10)	0	0	3 (50)	1 (17)	1 (17)

NOTE: N (%): count of patients (percentage per group); TEAE: treatment emergent adverse event

**Table 4:** Frequency of the most common adverse events during the multiple dose study

System organ class	Preferred term	Multiple Dose Study				
		
		Placebo	Ferroquine			
			
		(N = 7)	400 mg (N = 6)	600 mg (N = 6)	800 mg (N = 6)	1000 mg (N = 1)
**Any class**	Any TEAE	6 (86)	6 (100)	6 (100)	6 (100)	1 (100)

**Gastrointestinal disorders**	Any TEAE	4 (57)	3 (50)	4 (67)	5 (83)	1 (100)
	
	Abdominal pain	3 (43)	1 (17)	1 (17)	2 (33)	1 (100)
	
	Diarrhoea	0	0	0	2 (33)	0
	
	Nausea	1 (14)	2 (33)	2 (33)	3 (50)	0
	
	Toothache	1 (14)	1 (17)	0	2 (33)	0
	
	Vomiting	0	1 (17)	2 (33)	1 (17)	1 (100)

**system disorders**	Any TEAE	1 (14)	1 (17)	3 (50)	1 (17)	0
	
	Dizziness	0	0	1 (17)	0	0
	
	Headache	1 (14)	1 (17)	2 (33)	1 (17)	0

NOTE: N (%): count of patients (percentage per group); TEAE: treatment emergent adverse event

In the three-day repeated, ascending dose study, six patients out of seven in the placebo group experienced at least one adverse event during the trial versus all of the subjects taking ferroquine (19/19). The adverse events reported were mainly in the gastrointestinal tract, abdominal pain (16), diarrhoea (5), nausea (13), vomiting (9) and toothache (6), and in the nervous system disorders, headache (11) and dizziness (5), but also those that appeared during the follow-up period, in the musculoskeletal and connective tissue disorders (9), such as arthralgia and back pain, the injury, poisoning and procedural complications (10) (injury, post-traumatic pain, thermal burn).

Other symptoms such as mild, transient eye disorders (blurred vision, floating objects), mild skin rash (ECGs electrode application sites dermatitis or pruritis), infections and infestations, and fatigue were infrequent and were single occurrences across all dose groups in either study. All adverse events were considered mild to moderate in intensity and were transient. There were no deaths, serious adverse events, or adverse events leading to withdrawal reported during the trials.

Because of the small numbers of volunteers studied at each dose, no significant conclusions can be drawn from comparisons of adverse events between dose groups at the individual dose levels.

### Clinical laboratory evaluation

The clinical laboratory evaluation was performed through clinical chemistry and haematology tests of samples taken at different time points of scheduled visits until the end-of-study visit (60 days). The majority of the clinical chemistry potentially clinically significant abnormalities observed on patients treated with concerned elevations of creatine phosphokinase (≥3 ULN, normal range 25-90 IU). In most cases, creatine phosphokinase was elevated at baseline and was related to intense farming activities prior to inclusion (Table 5 and Table 6).

**Table 5:** Summary of participants with PCSA (parameters with PCSA definitions) for clinical chemistry and haematology parameters during the single dose study

Laboratory blood parameters (PCSA definition)	Normal range	Single Dose Study					
		
		Placebo	Ferroquine				
			
		(N = 10)	400 mg (N = 6)	800 mg (N = 6)	1200 mg (N = 6)	1400 mg (N = 6)	1600 mg (N = 6)
**Eosinophils (> max (0.5 Giga/L, ULN))**	0-1320/μl	6	5	1	3	6	5

**Haemoglobin (decrease**≥ **2 g/dL versus baseline)**	10.0-16.5 g/dL	1	0	4	1	0	0

**Neutrophils (<1.0 Giga/L, Black)**	1400-8140/μL	4	3	2	1	1	4

**Platelets (<100 Giga/L)**	120-400 Giga/L	0	0	0	0	0	0

**White Blood Cells (<2 Giga/L, Black)**	3.5-11/μL	0	1	1	0	0	0

**ALT (PCSA >2 ULN)**	< 45 U/L	0	1	1	0	0	0

**AST (PCSA >2 ULN)**	< 45 U/L	0	1	1	0	0	2

**Alkaline phosphatase (>1.5 ULN)**	13 - 100 U/L	0	0	1	0	0	0

**CPK (**≥ **3 ULN)**	25-90 U/L	1	0	2	2	1	1

**Creatinine (**≥ **150 μmol/L, adults)**	<133 μmol/L	1	0	1	0	0	1

**Gamma GT (**≥ **3 ULN)**	<50 U/L	0	0	1	0	0	0

**Glucose (≤ 2.5 mmol/L)**	3.6-6.1 mmol/L	0	0	0	0	1	1

**Glucose (**≥ **2 mmol/L, fasted)**	3.6-6.1 mmol/L	1	0	0	0	0	0

**Potassium (< 3 mmol/L)**	3.5-5.0 mmol/L	0	1	0	0	0	0

**Potassium (**≥ **5.5 mmol/L)**	3.5-5.0 mmol/L	0	0	0	0	1	1

**Total bilirubin (**≥ **34 μmol/L)**	0-17 μmol/L	1	1	0	0	0	0

**Total cholesterol (**≥ **7.74 mmol/L)**	120-240 mg/dL	0	0	0	0	0	0

**Triglycerides (**≥ **4.6 mmol/L)**	0-200 mg/dL	0	0	0	0	0	0

NOTE: N: count of patients; PCSA: Potentially Clinically Significant Abnormality (version 2.2, December 2004); LLN/ULN: Lower/Upper Limit of Normality. For hemoglobin, baseline values <LLN or >ULN are counted normal; for eosinophils, values <LLN are counted as normal. For ALT, AST, ALP, Total Bilirubin, GGT, CPK, baseline values <LLN are counted normal

**Table 6:** Summary of participants with PCSA (parameters with PCSA definitions) for clinical chemistry and haematology parameters during the multiple dose study

Laboratory blood parameters (PCSA definition)	Normal range	Multiple Dose Study				
		
		Placebo	Ferroquine			
			
		(N = 7)	400 mg (N = 6)	600 mg (N = 6)	800 mg (N = 6)	1000 mg (N = 1)
**Eosinophils (> max (0.5 Giga/L, ULN))**	0-1320/μl	4	2	4	4	1

**Haemoglobin (decrease**≥ **2 g/dL versus baseline)**	10.0-16.5 g/dL	0	0	0	0	0

**Neutrophils (<1.0 Giga/L, Black)**	1400-8140/μL	0	3	0	2	0

**Platelets (<100 Giga/L)**	120-400 Giga/L	1	1	2	0	0

**White Blood Cells (<2 Giga/L, Black)**	3.5-11/μL	0	1	0	0	0

**ALT (normal <45UI, PCSA >2 ULN)**	< 45 U/L	1	1	2	1	0

**AST (normal <45UI, PCSA >2 ULN)**	< 45 U/L	1	1	0	1	0

**Alkaline phosphatase (>1.5 ULN)**	13 - 100 U/L	0	1	0	1	0

**CPK (**≥ **3 ULN)**	25-90 U/L	3	4	5	5	1

**Creatinine (**≥ **150 μmol/L, adults)**	<133 μmol/L	0	1	0	0	0

**Gamma GT (**≥ **3 ULN)**	<50 U/L	0	1	0	1	0

**Glucose (≤ 2.5 mmol/L)**	3.6-6.1 mmol/L	0	0	0	0	0

**Glucose (**≥ **2 mmol/L, fasted)**	3.6-6.1 mmol/L	0	0	0	0	0

**Potassium (< 3 mmol/L)**	3.5-5.0 mmol/L	0	0	0	0	0

**Potassium (**≥ **5.5 mmol/L)**	3.5-5.0 mmol/L	2	0	2	0	0

**Total bilirubin (**≥ **34 μmol/L)**	0-17 μmol/L	1	1	0	0	0

**Total cholesterol (**≥ **7.74 mmol/L)**	120-240 mg/dL	0	0	0	0	0

**Triglycerides (**≥ **4.6 mmol/L)**	0-200 mg/dL	0	0	0	0	0

NOTE: N: count of patients; PCSA: Potentially Clinically Significant Abnormality (version 2.2, December 2004); LLN/ULN: Lower/Upper Limit of Normality. For hemoglobin, baseline values <LLN or >ULN are counted normal; for eosinophils, values <LLN are counted as normal. For ALT, AST, ALP, Total Bilirubin, GGT, CPK, baseline values <LLN are counted normal

In the single dose study, five patients out of 40 showed an alanine transaminase increase >1 ULN (defined as 45 IU/ml) at any time point of the study with a possible association to changes of other liver functions. Among them were four patients treated with ferroquine and one patient treated with placebo. These changes were asymptomatic and, therefore, were not reported as adverse events. One patient under placebo experienced an increased level of alanine transaminase, with a maximum value of 59 IU on Day 21. The increase was associated to a parallel and moderate increase of total bilirubin (19 μmol/L, normal range <17 μmol/L) and a moderate increase of aspartate transaminase and gamma glutamyl transferase. In the 800 mg dose-group, one patient had an increased level of alanine transaminase with a maximum value of 148 IU on Day 15, with associated increases in total bilirubin (24 μmol/L), aspartate transaminases, alkaline phosphatases, and gamma glutamyl transferase. In the 1,200 mg dose-group, at one patient an increased level of alanine transaminase with a maximum value of 52 IU was observed on Day 7, whereas, alkaline phosphatase and total bilirubin (5 μmol/L) remained within normal ranges. At one patient treated with 1,400 mg, an increased level of alanine transaminase with a maximum value of 47 IU was observed on Day 15, associated to parallel changes in other liver function parameters at the exception of the bilirubin level (8 μmol/L). Finally, one patient was one of the 1,600 mg dose-group experienced an increase of alanine transaminase values with a maximum value of 48 IU on Day 2 with parallel increase in total bilirubin (25 μmol/L) on Day 15. The respective parameters of this patient were already slightly elevated at baseline (Day -1).

In the three-day repeated dose study, elevated alanine transaminases (>2 ULN) was observed in one placebo-treated patient and in four patients in the ferroquine groups. One patient in the 400 mg group (normal baseline 0.38 ULN) had an alanine transaminase >10 ULN starting at Day 12 with a maximum magnitude of 16.55 ULN, which was associated to abdominal pain and headache. Over the course of the following week, alkaline phosphatases and bilirubin increased and picked at about 2 ULN and 1.7 ULN, respectively. Infectious causes were ruled out. However, the patient reported intake of an herbal tea against abdominal pain, which might have contributed to the enzyme abnormalities. Alanine transaminases returned to normal by Day 37 (0.71 ULN). A patient of the 600 mg group, who had a slightly elevated alanine transaminase value at screening (1.22 ULN), but within normal ranges at baseline (0.91 ULN), presented again an elevated value on Day 3 (1.71 ULN) and reached the potentially clinically significant abnormality level (2.18 ULN) on Day 5, subsequently followed by a decrease to normal rangeby Day 12. In this same 600 mg dose group, another patient with normal baseline value of 0.42 ULN, presented an elevated alanine transaminase values on Day 3 that reached the potentially clinically significant abnormality level on Day 23 (2.56 ULN). His values were still elevated at the end-of-study visit (1.78 ULN). Finally, one patient of the 800 mg dose group, with normal baseline value of 0.38 ULN, had a peak alanine transaminases value of 6.37 ULN and a bilirubin value of 1.48 ULN at Day 12 without clinical symptoms. Alkaline phosphatases peaked at 1.77 ULN on Day 17. Liver parameter values decreased toward the normal range by the end-of-study visit. The most frequently observed haematology, potencially clinically significant abnormalities were related to eosinophils that were likely to be related to concomitant parasitic infections existing at baseline.

### Electrocardiogram evaluation

In the single dose study, no QTcF values >450 ms, or delta QTcF >60 ms were observed in any treatment group. One patient in the 1,200 mg dose group experienced a delta QTcF value between 30 and 60 ms, while a moderate change in mean increases from baseline of + 8.2 ms was observed for the 1,600 mg dose level coinciding with the time of maximal plasma concentrations. In the three-day repeated dose study, continuous monitoring did not show any clinically significant arrhythmic episode developing after drug administration in any patient. A deviation in the repolarization pattern (inversion of T wave), visible from T5 upwards and lasting for several hours, was observed in four patients of the 800 mg dose group, especially in one patient who showed a clear and long-lasting T inversion each day of investigational product administration. According to previous experiences and the known influence of aminoquinolines on ECGs, these episodes were considered to be related to the investigational product, but remained without any clinical significance. In the 800 mg dose group, there were 12.6 ms, 16.8 ms, and 19.2 ms mean increases in QTcF intervals on Day 3 at 6, 8, and 12 hours, respectively. Additionally, in the 800 mg dose group, there was a 19.6 ± 6.8 ms (mean ± SEM) maximum mean increase in QTcF intervals, compared to maximum mean increases of 9.9 ± 5.5, 5.2 ± 3.6, and 16.5 ± 6.8 ms for the placebo, 400 mg, and 600 mg groups, respectively. There were no delta QTcF intervals >60 ms and no QTcF intervals >450 ms.

## Discussion

Despite an increase in international investment in anti-malarial drug development in the past decade only a few novel drugs have been developed [12-19]. Ferroquine is one of the few novel anti-malarials entering clinical development. A favourable safety and tolerability profile of ferroquine could be demonstrated when administered as a single dose up to 1,600 mg or as three consecutive daily doses up to 800 mg. No dose limiting clinical adverse event was observed at the investigated dose levels. All adverse events were mild to moderate, transient, and no serious drug related adverse event was observed. These findings are in line with previous data of preclinical studies and the first in human study performed in healthy Caucasian subjects [unpublished data].

Gastrointestinal side effects and central nervous disorders appear to be the most common clinical adverse events following treatment with ferroquine. These symptoms were however self-limiting and mild to moderate in intensity. Most important the frequency of these events was similar in patients treated with ferroquine and those receiving placebo and overall compared well to currently used anti-malarials. However, these side effects are well described for other 4-aminoquinoline anti-malarials and need, therefore, further investigation in the case of ferroquine [20-26]. Other less common adverse events, such as fatigue, blurred vision and rash were mild, transient, and were single occurrences across all dose groups.

One limitation of the assessment of adverse events in this study may be the potential progression of asymptomatic infection to malaria. However, the inclusion of a control group with placebo treatment helped to minimize this factor in the absence of knowledge on the efficacy of ferroquine in humans. Similarly, the apparent dose dependent increase in gastrointestinal adverse events may be either due to pharmacodynamic properties of the drug or alternatively to the high number of capsules that had to be swallowed (up to 16 capsules per participant). In summary, ferroquine may lead to gastrointestinal side effects comparable to other anti-malarials. However, the number of participants in this study does not permit to make a final judgement on a potential dose dependent increase of gastrointestinal toxicity of ferroquine.

At eight weeks follow-up, there was no evidence for cardiac, ocular, hepatic, haemotologic, renal, dermatologic, or other end-organ adverse events. Although adverse events involving these and other organs have been reported with aminoquinolines previously [21,23-25], these findings have typically been observed in persons treated for prolonged periods of time (more than 5 years) at doses 200-400 mg base or higher per day [21,27]. The absence of clinically significant detectable adverse events and the normal laboratory tests in all volunteers at eight weeks of follow-up are consistent with previous reports on the safety of short-term chloroquine treatment [20,21,26].

The finding of abnormal liver function tests in the course of the study. These observations are consistent with data from toxicology studies on rats and dogs which showed small increases of alanine transaminase and aspartate aminotransferase (unpublished data). These changes were minor and not considered to be of toxicological significance. In our clinical trials the laboratory changes were not clinically significant and most of the abnormal test results normalized during the course of the study without further intervention. Important is that no linear dose depending relationship between the administration of ferroquine and increase of liver parameters was observed in this study.

Based on previous animal and human studies showing prolongation of the QT interval for chloroquine a special emphasis was laid on the assessment of cardiac effects [28-32]. Ferroquine itself has been shown to have in vitro effects on hERG channels. There was also a trend toward changes in QTc interval parameters in telemetered animals. The current findings show several patients in the repeated dose study with T wave morphology changes on their ECG readings following ferroquine administration, and most of the time associated with U waves. These findings even though they are not clinically significant highlight the potential of a cardiac effect of ferroquine on humans.

## Conclusions

In conclusion, ferroquine showed a favourable tolerability and safety profile up to 1,600 mg when administered as single dose, and appears to be well tolerated up to 800 mg once daily for three days. Although gastrointestinal disorders (including nausea and vomiting) and nervous system disorders (including dizziness and headache) appear to be the prevalent clinical adverse events, the liver is the potential target organ and it should be carefully monitored via liver function tests. Ferroquine has the potential to prolong the QTc interval and affect T wave morphology. Subjects should undergo careful ECG monitoring while taking this investigational product.

## Competing interests

Authors declare no conflict of interest. This study was funded by the Department of Research of Sanofi-Aventis as part of the developing programme of ferroquine. Part of this work was presented as dissertation by GMN for his medical degree's thesis at the Faculty of Medicine in Libreville, Gabon, MK, PGK and BL were co-directors of the thesis (Université des Sciences de la Santé (USS), Thesis n°524, 2006)

## Authors' contributions

GMN carried out the safety study and drafted the manuscript. CS, MDB, MAM, PBM, COS, BL carried out the study. MK, MR, PGK participated in the design of the study and performed the statistical analysis. DTM and BL conceived of the study, and participated in its design and coordination. All authors read and approved the final manuscript.
